# Peptidomic evidence for pH-stat flavourzyme-mediated debittering of Alcalase-derived rice protein hydrolysates

**DOI:** 10.1016/j.crfs.2026.101529

**Published:** 2026-08-10

**Authors:** Pei-Yu Wu, Yu-Shun Lin, Mei-Ling Li, Yun-Hsuan Chien, Shang-Ming Huang, Kuo-Chiang Hsu

**Affiliations:** a Department of Nutrition, China Medical University, No.100, Sec.1, Jingmao Rd., Beitun Dist., Taichung City, 406040, Taiwan; b Department of Food Nutrition and Health Biotechnology, Asia University, 500 Lioufeng Rd., Wufeng, Taichung, 41354, Taiwan

**Keywords:** Rice protein, Enzymatic hydrolysis, Debittering, Flavourzyme, Peptidomics, Q value, pH control mode, Peptide profile, Bitterness

## Abstract

Flavourzyme-assisted two-stage hydrolysis is a recognized debittering strategy for protein hydrolysates, yet peptidomic-level evidence for the underlying mechanism—particularly the role of pH control mode—remains limited. This study compared pH-stat and free-fall pH Flavourzyme treatments (3 and 4 h) of an Alcalase-derived rice protein hydrolysate (A6; degree of hydrolysis by the o-phthalaldehyde method [DH-OPA] 24.69%) using nanoLC-ESI-Q-TOF de novo sequencing to characterize peptide-profile changes associated with bitterness reduction.

Formal sensory evaluation (15 trained panelists, paired comparison with Bonferroni correction for 10 comparisons) of the ultrafiltered (<2500 Da) fractions placed A6-s3 (pH-stat, 3 h) at the least-bitter end of the ranking, although it did not differ significantly from A6-s4 after multiplicity correction. The apparent ranking differed from that observed in the preliminary evaluation of unfiltered hydrolysates, which used a smaller panel and a different sensory design. De novo sequencing identified 963–1226 peptides per hydrolysate. A6-s3 exhibited the lowest mean Q value (1430 cal mol^−1^; Kruskal–Wallis, p < 10−22) and the lowest predicted bitter peptide proportion (49.6%). A6 and A6-s3 shared only one exact PEAKS-assigned sequence among >1900 combined detections, indicating extensive differences in the detectable peptide profiles. Database-assisted annotation against the *Oryza sativa* proteome showed a markedly lower assignment rate for A6-s3 (0.2%) than for A6 (52.8%, predominantly glutelins); this was treated as a descriptive analytical observation because I/L ambiguity, de novo sequencing uncertainty, stochastic data-dependent acquisition (DDA) sampling, and database coverage may contribute. A6-s3 also showed the lowest C-terminal strongly hydrophobic residue frequency (62.3%; I, L, F, W, Y, P) and the highest N-terminal amino acid diversity (Shannon entropy, 3.91 bits).

These findings indicate that maintaining pH during Flavourzyme treatment was associated with a more favorable bitterness-related peptide profile. The results are compatible with established terminal processing accompanied by broader changes in the detectable peptide population, but they do not establish or quantify a distinct contribution from internal cleavage.

## Highlights

• pH-stat Flavourzyme treatment for 3 h placed A6-s3 at the least-bitter end.
• A6-s3 showed the lowest mean Q value and predicted bitter peptide proportion.
• A6 and A6-s3 shared only one exact sequence among >1900 combined detections.
• A6-s3 showed reduced C-terminal strongly hydrophobic residue frequency.
• pH maintenance was associated with broader changes in the detectable peptide profile.

## Graphical abstract

**Figure:**
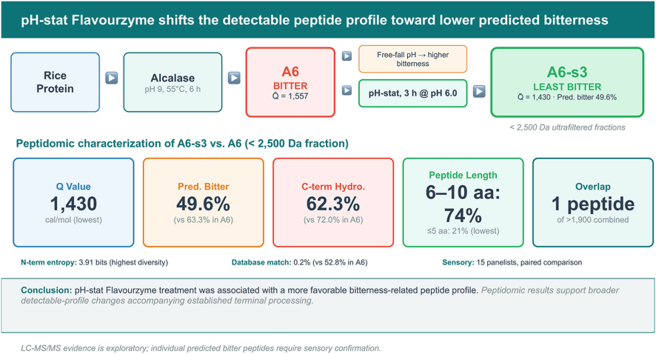

## 1 Introduction

Rice protein is a promising plant-based protein source owing to its hypoallergenicity, balanced amino acid profile, and wide availability (Shih, 2003). However, native rice protein has poor solubility at neutral pH because of extensive disulfide cross-linking and hydrophobic aggregation, restricting its use in beverages and liquid food products (Amagliani et al., 2017; Zhao et al., 2012).

Enzymatic hydrolysis effectively improves protein solubility by disrupting compact protein structures and releasing soluble peptides (Klompong et al., 2007). Among commercial proteases, Alcalase is particularly suited to rice protein owing to its broad specificity under alkaline conditions (Hamada, 2000). However, extensive hydrolysis can generate short hydrophobic peptides that elicit pronounced bitterness, and the sensory outcome depends on enzyme specificity and the resulting peptide population rather than degree of hydrolysis alone (Saha and Hayashi, 2001; FitzGerald and O'Cuinn, 2006; Groβmann et al., 2021; Fan et al., 2019).

Two-stage hydrolysis with Flavourzyme, a fungal protease/peptidase complex from *Aspergillus oryzae* possessing both endo- and exopeptidase activities, has been proposed to reduce bitterness (Liu et al., 2022; Meinlschmidt et al., 2016). Exopeptidase-assisted debittering through further processing of terminal hydrophobic residues is an established mechanism in food protein hydrolysates (Saha and Hayashi, 2001; FitzGerald and O'Cuinn, 2006; Groβmann et al., 2021; Fu et al., 2019; Ewert et al., 2018). The prevailing explanation is that terminal processing lowers average peptide hydrophobicity (Q value), potentially shifting peptides below the 1400 cal mol^−1^ threshold proposed by Ney (Adler-Nissen, 1986; Ney, 1971). Nevertheless, sensory outcome is influenced by the combined actions of endo- and exopeptidases and by the resulting peptide population (Groβmann et al., 2021; Fu et al., 2019; Xia et al., 2022). Population-level peptidomic evidence comparing peptide profiles before and after Flavourzyme treatment remains limited, and direct comparison of pH-stat and free-fall processing modes has received little attention.

A further factor that may influence the debittering outcome is the pH control mode. In pH-stat hydrolysis the reaction pH is maintained at the enzyme's optimum by continuous alkali titration, whereas in free-fall pH mode the pH decreases naturally as hydrolysis proceeds (Ney and Boudreau, 1979). Although pH-stat is routinely used for DH measurement, its effects on peptide profiles and sensory bitterness have rarely been compared directly with those of free-fall pH under otherwise matched conditions.

Beyond Ney's Q-value rule, peptide bitterness is modulated by sequence length, terminal residue identity, charge distribution, and sequence context (Maehashi and Huang, 2009; Ishibashi et al., 1988). Sequence-based tools such as iBitter-SCM and BERT4Bitter incorporate residue composition and local sequence information beyond mean hydrophobicity, while BitterPredict illustrates broader machine-learning approaches to bitterant classification (Charoenkwan et al., 2020, 2021; Dagan-Wiener et al., 2017). Sensory–peptidomic approaches have recently been applied to selected food systems, including enzymatically debittered cheese flavoring, but comparable population-level analyses in rice protein hydrolysates remain scarce (Xiang et al., 2024; Dallas et al., 2015). Rice-protein oligopeptides are increasingly studied for their preparation, bioactivity, debittering, and application potential (Yan et al., 2024). Accordingly, this study aimed to (1) generate a soluble Alcalase-derived rice protein hydrolysate as a model bitter substrate; (2) determine how pH-stat and free-fall Flavourzyme treatment affect sensory bitterness; and (3) characterize associated changes in the detectable peptide profile using de novo sequencing, database-assisted annotation, Q-value analysis, peptide-length distribution, terminal-residue characteristics, and peptide-overlap mapping.

## 2 Materials and methods

### 2.1 Materials and reagents

Rice protein concentrate (protein content 83.7%, dry basis; Kjeldahl, N × 5.95) was obtained from a local supplier (Taichung, Taiwan). Alcalase 2.4 L (EC 3.4.21.62, ≥ 2.4 AU g^−1^) and Flavourzyme 1000 L (EC 3.4.11.1, ≥ 1000 LAPU g^−1^) were from Novozymes (Bagsværd, Denmark). Papain (EC 3.4.22.2, ≥ 3.0 AU mg^−1^), o-phthalaldehyde (OPA), 2,4,6-trinitrobenzenesulfonic acid (TNBS), L-leucine, L-leucyl-glycine, bovine serum albumin (BSA), and sodium dodecyl sulfate (SDS) were from Sigma-Aldrich (St. Louis, MO, USA). All other chemicals were of analytical grade.

### 2.2 First-stage hydrolysis

**Fig. 1:**
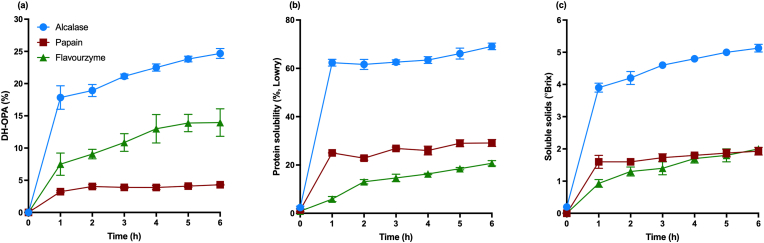
Time course of first-stage hydrolysis of rice protein with three proteases: (a) DH-OPA, (b) protein solubility (Lowry), and (c) soluble solids. Conditions: Alcalase (pH 9, 55 °C), Papain (pH 7, 50 °C), Flavourzyme (pH 6, 60 °C); E/S = 5%, pH-stat. Values are mean ± SD (n = 3).

Rice protein was suspended in distilled water at 1:19 (w/w) and hydrolyzed with Alcalase (pH 9, 55 °C), papain (pH 7, 50 °C), or Flavourzyme (pH 6, 60 °C) at an enzyme-to-substrate ratio (E/S) of 5% (w/w) under pH-stat conditions. Hydrolysis was conducted for 6 h with stirring at 120 rpm. Aliquots were withdrawn at 0–6 h, heated at 95 °C for 15 min, and centrifuged at 8000 × g for 20 min. Based on the results (Fig. 1, Table S4), Alcalase at 6 h (designated A6) was selected as the substrate for second-stage hydrolysis.

### 2.3 Second-stage hydrolysis

The A6 supernatant was hydrolyzed with Flavourzyme (E/S = 5%, pH 6, 60 °C) under two pH control modes: (1) pH-stat, with pH maintained at 6.0 by continuous addition of 2 N NaOH; and (2) free-fall pH, with no pH adjustment after the initial setting. For each mode, hydrolysis was conducted for 3 or 4 h, giving four conditions: A6-f3 (free-fall, 3 h), A6-f4 (free-fall, 4 h), A6-s3 (pH-stat, 3 h), and A6-s4 (pH-stat, 4 h). In free-fall mode, the pH decreased from 6.0 to 5.5 (1 h), 5.4 (2 h), 5.2 (3 h), and 5.1 (4 h). After enzyme inactivation (95 °C, 15 min) and centrifugation, the supernatants were ultrafiltered through a 2500 Da MWCO membrane (Amicon Ultra, Millipore, Burlington, MA, USA). The permeates were freeze-dried and reconstituted to an equivalent nitrogen concentration, as determined by the Kjeldahl method, before sensory evaluation. The same ultrafiltered peptide preparations were subsequently used for LC-MS/MS analysis.

### 2.4 Degree of hydrolysis

DH was assessed using the OPA method with L-leucyl-glycine as the standard (Nielsen et al., 2001) and the TNBS method with L-leucine as the standard (Adler-Nissen, 1979) for all hydrolysates. For reactions conducted under pH-stat conditions, DH was additionally calculated from cumulative NaOH consumption (Adler-Nissen, 1986). DH-OPA served as the primary indicator for comparisons across pH-control modes because it was applicable to both pH-stat and free-fall treatments.

### 2.5 Protein solubility and soluble solids

Protein solubility was determined by the Kjeldahl method (N × 5.95) and the Lowry method with BSA as standard (Lowry et al., 1951). Soluble solids were measured with a digital refractometer (Atago PAL-1, Tokyo, Japan) and expressed as °Brix.

### 2.6 Sensory evaluation

This study was approved by the Research Ethics Committee of China Medical University and Hospital (CMUH114-REC1-034, approved March 20, 2025). All panelists provided written informed consent.

A preliminary evaluation was conducted with 5 trained panelists to select hydrolysis time endpoints. Using A6 (score = 10, most bitter) and distilled water (score = 0) as anchors, panelists rated the bitterness of 8 secondary hydrolysates (f1–f4 and s1–s4, without ultrafiltration) on a 0–10 scale. These results were used only for time-point screening, not for final ranking. Based on the results, f3, f4, s3, and s4 were selected for formal evaluation.

Formal evaluation was conducted with 15 panelists using a paired-comparison design following established sensory principles for blinding, randomization, and balanced presentation (Lawless and Heymann, 2010). Five ultrafiltered hydrolysates (A6, A6-f3, A6-f4, A6-s3, A6-s4), reconstituted to equivalent nitrogen concentration, were compared in all 10 pairwise combinations across two sessions. Panelists rated the degree of bitterness difference on a 0–7 scale (0 = no difference, 7 = extremely different) and indicated which sample was more bitter. The bitterness ranking was derived by three-stage comparison: (1) each secondary hydrolysate vs. A6; (2) among secondary hydrolysates; (3) between pH control modes at the same duration. Significance was assessed by the Wilcoxon signed-rank test for pairs with difference scores and the sign test for pairs with direction-only data, with Bonferroni correction for 10 comparisons. Full details of the sensory evaluation design, an example ballot, and pairwise statistical results are provided in Table S7–S8 and Fig. S1.

### 2.7 LC-MS/MS analysis

Ultrafiltered peptide samples were analyzed at the Proteomics Core Laboratory, China Medical University, using a nanoUHPLC system (UltiMate 3000 RSLCnano, Dionex, Sunnyvale, CA, USA) coupled to a quadrupole time-of-flight (Q-TOF) mass spectrometer (maXis Impact, Bruker Daltonics, Bremen, Germany) via electrospray ionization (ESI). Separation was performed on an Acclaim PepMap C18 column (75 μm × 250 mm, 2 μm, 100 Å; Thermo Scientific) at a flow rate of 300 nL min^−1^ with mobile phases of 0.1% formic acid in water (A) and 0.1% formic acid in acetonitrile (B). The gradient was 8–40% B over 90 min (0.35% min^−1^). Mass spectra were acquired in positive ion mode (m/z 150–1500) with data-dependent acquisition (DDA); the top 8 precursor ions carrying +2, +3, or +4 charges were selected for MS/MS fragmentation. Scan rates were 1 Hz for MS1 and 10 Hz for MS2. Complete acquisition and data analysis parameters are listed in Table S9.

Prior to analysis, peptide samples were reduced with 10 mM dithiothreitol (56 °C, 30 min) and alkylated with 55 mM iodoacetamide (room temperature, 30 min in the dark). Equal amounts of peptide material were injected for LC-MS/MS analysis. De novo sequencing and database search were performed using PEAKS Studio 11.0 (Bioinformatics Solutions Inc., Waterloo, ON, Canada) with parent mass tolerance 50 ppm and fragment mass tolerance 0.05 Da. Fixed modification was set to carbamidomethylation (C); variable modifications included oxidation (M) and deamidation (NQ). The database search was conducted against the *Oryza sativa* (rice) protein sequences from UniProt/SwissProt (accessed June 2024) with unspecific enzyme cleavage. Peptides were considered database-matched when PEAKS assigned at least one *Oryza sativa* protein accession under the specified search settings. Only de novo peptide tags with an average local confidence (ALC) score ≥ 75% were retained; all identified peptides in the present study met this criterion (minimum ALC 75.8–86.2% across samples). Because de novo sequencing cannot distinguish the isobaric residues isoleucine (I) and leucine (L), all I/L assignments should be considered interchangeable, and the peptide overlap analysis (Fig. 6) was performed using exact sequence matches as assigned by PEAKS. Peptide peak areas were obtained from the PEAKS feature detection module; for peptides detected in multiple scans, areas were summed. Sensitivity analysis showed that A6-s3 retained the lowest mean Q value and the lowest predicted bitter peptide proportion at ALC cutoffs of 80% and 90%; peptide overlap between A6 and A6-s3 remained ≤1 across all cutoffs (Table S5).

### 2.8 Bitterness prediction by Q value

The average hydrophobicity (Q value) of each peptide was calculated as Q = ΣΔf/n, where Δf is the side-chain free energy of transfer from ethanol to water (cal mol^−1^) and n is the number of residues, using side-chain hydrophobicity values from Tanford (1962) as applied by Ney (1971). Peptides with Q > 1400 cal mol^−1^ were classified as predicted bitter. Abundance-weighted Q values were calculated as Σ(Q_i_ × Area_i_)/Σ(Area_i_), where Area_i_ is the LC-MS/MS peak area.

### 2.9 Terminal amino acid analysis

N-terminal and C-terminal amino acid frequencies were calculated for each hydrolysate. Higher-Δf residues were defined according to the Ney/Tanford side-chain transfer free-energy scale as V, I, K, L, M, F, Y, W, and P (Δf ≥ 1300 cal mol^−1^); strongly hydrophobic residues were defined as I, L, F, W, Y, and P (Δf ≥ 2400 cal mol^−1^). Terminal diversity was quantified by Shannon entropy H = −Σp_i_ log2 p_i_, where p_i_ is the frequency of amino acid i at the terminal position.

### 2.10 Total amino acid composition

Total amino acid composition was determined by an external testing laboratory using in-house methods based on AOAC Official Method 994.12 for amino acids in feeds; tryptophan was determined separately using a method based on AOAC Official Method 988.15 (AOAC International, 2005). Results were expressed as g kg^−1^ hydrolysate. Because one determination was available for each sample, these data were interpreted descriptively and were not used for inferential statistical analysis or to estimate free-amino-acid concentrations.

### 2.11 Statistical analysis

Results are expressed as mean ± SD. One-way ANOVA with Fisher's LSD post-hoc test was used for physicochemical data. The Kruskal–Wallis H test with Dunn's post-hoc test (Bonferroni correction) was used for Q value distributions. The chi-square test with Bonferroni correction was used for predicted bitter peptide proportions. Sensory paired comparisons were analyzed by the Wilcoxon signed-rank test (for pairs with difference scores) or the sign test (for pairs with direction-only data), with Bonferroni correction for 10 comparisons. Significance was set at p < 0.05. Analyses were performed in GraphPad Prism 11 (GraphPad Software, San Diego, CA, USA) and Python 3.12 (SciPy 1.14). Because LC-MS/MS analysis was performed without biological replicate acquisitions, individual detected peptides were treated as observational units for exploratory distribution-level comparisons. The resulting p values describe differences among the peptide sets detected in the present runs and should not be interpreted as treatment-level biological replication. No dedicated quality-control injections were performed; intra-batch technical variability was therefore not formally assessed. Accordingly, the peptidomic results should be interpreted as exploratory observations derived from a single DDA acquisition per sample rather than as a formally validated quantitative peptidomic dataset.

## 3 Results and discussion

### 3.1 First-stage protease selection

Among the three proteases, Alcalase yielded significantly higher DH-OPA (24.69 ± 0.77%), protein solubility (78.12 ± 3.02%, Kjeldahl), and soluble solids (5.13 ± 0.12 °Brix) than papain and Flavourzyme after 6 h (p < 0.05; Fig. 1, Table S4). The complete time-course data for all indicators are provided in Table S4. The superior performance of Alcalase is attributable to its broad cleavage specificity under alkaline conditions, which promotes unfolding and extensive hydrolysis of rice protein (Hamada, 2000). In particular, the markedly higher Kjeldahl-based protein solubility of Alcalase hydrolysates (78% vs. 32% for papain and 26% for Flavourzyme) made Alcalase the most suitable first-stage protease for generating a soluble substrate for subsequent Flavourzyme treatment. A6 was therefore selected as the substrate for second-stage debittering.

### 3.2 Second-stage hydrolysis: physicochemical properties

**Fig. 2:**
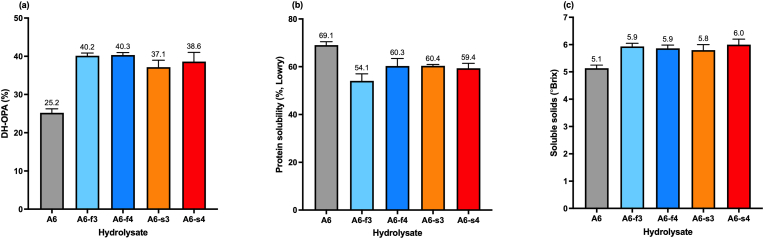
Physicochemical properties of rice protein hydrolysates after two-stage Flavourzyme hydrolysis: (a) DH-OPA, (b) protein solubility (Lowry), and (c) soluble solids. A6, Alcalase 6 h; f, free-fall pH; s, pH-stat; 3 and 4, duration (h). Values are mean ± SD (n = 3).

Flavourzyme treatment increased DH-OPA from 24.69% (A6) to 37.14–40.35% across all four conditions (Fig. 2a, Table 1), confirming continued peptide-bond cleavage. Kjeldahl-based solubility remained at 77–80% (Table 1), whereas Lowry-based solubility decreased from 69.10% to 54.08–60.37%. The discrepancy likely reflects the different analytical principles of the two methods. Kjeldahl quantifies total soluble nitrogen, whereas the Lowry response depends on peptide bonds and selected amino acid side chains and may vary with peptide size and composition (Ney, 1971; Spellman et al., 2003). Progressive hydrolysis can therefore alter the Lowry response without a corresponding decrease in total soluble nitrogen. Soluble solids increased slightly from 5.13 to 5.80–6.00 °Brix (Fig. 2c), indicating that the total soluble material was not substantially altered by second-stage hydrolysis.

**Table 1:** Physicochemical properties of rice protein hydrolysates after two-stage hydrolysis with Flavourzyme.

Sample	DH-OPA (%)	Solubility-Lowry (%)	Solubility-Kjeldahl (%)	Soluble solids (°Brix)	Yield (%)
**A6**	24.69 ± 0.77	69.10 ± 1.37	78.12 ± 1.18	5.13 ± 0.12	25.38
**A6-f3**	40.15 ± 0.70	54.08 ± 2.93	78.79 ± 0.56	5.93 ± 0.12	29.65
**A6-f4**	40.35 ± 0.64	60.30 ± 3.15	77.37 ± 1.50	5.87 ± 0.12	34.44
**A6-s3**	37.14 ± 1.84	60.37 ± 0.62	77.87 ± 1.24	5.80 ± 0.20	30.46
**A6-s4**	38.61 ± 2.41	60.21 ± 2.07	79.79 ± 1.63	6.00 ± 0.20	33.49

Values are mean ± SD (n = 3).

A6, Alcalase hydrolysate (6 h); f, free-fall pH; s, pH-stat; 3 and 4, Flavourzyme hydrolysis duration (h).

All samples were ultrafiltered (UF < 2500 Da) before analysis.

### 3.3 Apparent differences in bitterness rankings between preliminary and formal evaluations

**Fig. 3:**
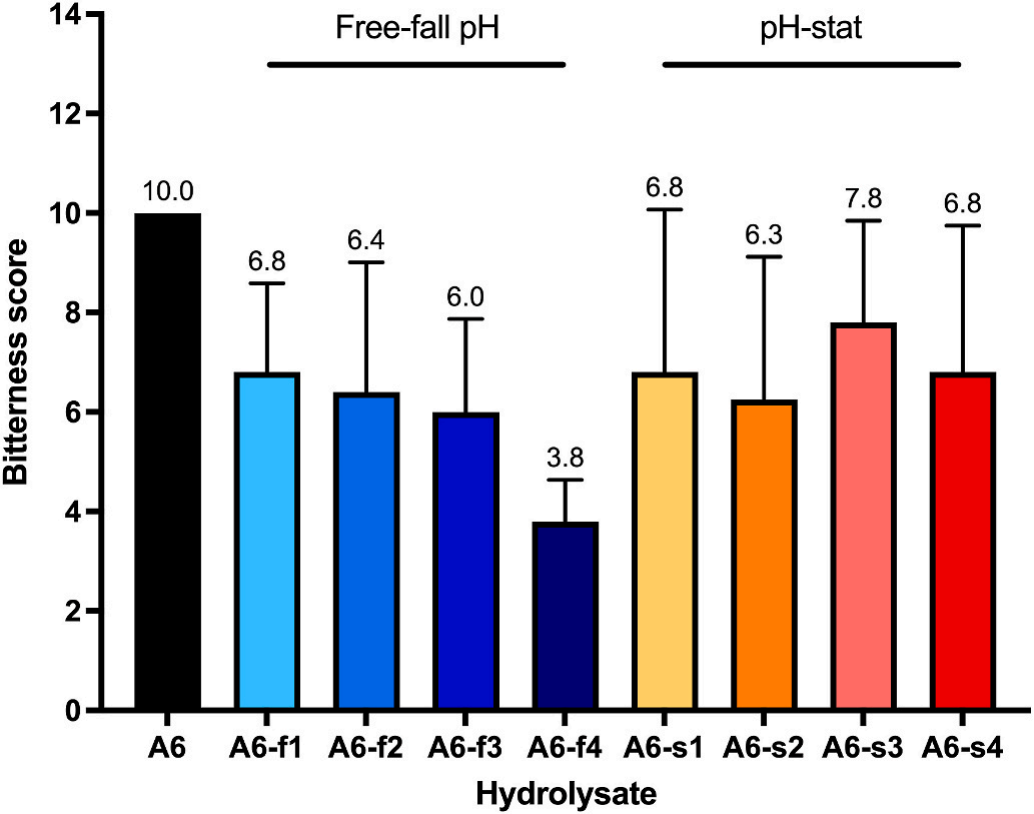
Preliminary bitterness screening of eight secondary hydrolysates before ultrafiltration. Scale: 0 (no bitterness) to 10 (most bitter = A6). These results were used for time-point selection only and do not represent the final bitterness ranking. Values are mean ± SD (n = 5 panelists).

In the preliminary evaluation without ultrafiltration (Fig. 3), bitterness decreased with increasing hydrolysis time under free-fall pH (f1: 6.8 ± 1.8 → f4: 3.8 ± 0.8; mean ± SD, n = 5), with A6-f4 being the least bitter and A6-s3 the most bitter (7.8 ± 2.0). These results were used solely for selecting time endpoints (3 and 4 h) and were not used for final ranking.

After ultrafiltration (<2500 Da), formal paired-comparison evaluation placed A6-s3 at the least-bitter end of the sensory ranking (Table 2). Eight of the 10 pairwise comparisons were significant after Bonferroni correction, whereas A6-f3 versus A6-s4 and A6-s3 versus A6-s4 were not significant (Table S8). Three observations emerged: (1) all four secondary hydrolysates were less bitter than A6; (2) under free-fall conditions, the 3-h sample was less bitter than the 4-h sample; and (3) pH-stat treatment produced less-bitter samples than free-fall treatment at the corresponding durations. Although A6-s3 occupied the least-bitter end of the ranking, it was not significantly different from A6-s4 after multiplicity correction. The apparent difference between preliminary and formal rankings may reflect changes in the sample matrix after ultrafiltration, including differential transmission of peptide-size populations. However, the preliminary and formal evaluations also differed in panel size (5 vs. 15 panelists) and methodology (anchored intensity scale vs. paired comparison). Because ultrafiltration status, panel size, and sensory methodology changed simultaneously, an ultrafiltration × sample interaction could not be statistically estimated from the present design. Consequently, the observed ranking difference cannot be attributed exclusively to ultrafiltration, and its mechanistic basis remains a working hypothesis.

**Table 2:** Pairwise degree of bitterness difference among rice protein hydrolysates (formal sensory evaluation).

	A6	A6-f3	A6-f4	A6-s3	A6-s4
**A6**	—	3.75*	2.78*	3.64*	4.03*
**A6-f3**	3.75*	—	3.33*	1.68*	ns
**A6-f4**	2.78*	3.33*	—	2.75*	2.13*
**A6-s3**	3.63*	1.68*	2.75*	—	ns
**A6-s4**	4.03*	ns	2.13*	ns	—

Scale: 0 (no difference) to 7 (extremely different). n = 15 panelists, two sessions.

*p < 0.05; ns, not significant (*p* > 0.05). Significance was assessed by the Wilcoxon signed-rank test for pairs with difference scores and the sign test for direction-only pairs, with Bonferroni correction for 10 comparisons.

All samples were ultrafiltered (UF < 2500 Da) before evaluation.

A6-s3 was located at the least-bitter end of the ranking but did not differ significantly from A6-s4.

### 3.4 Q value distributions

**Fig. 4:**
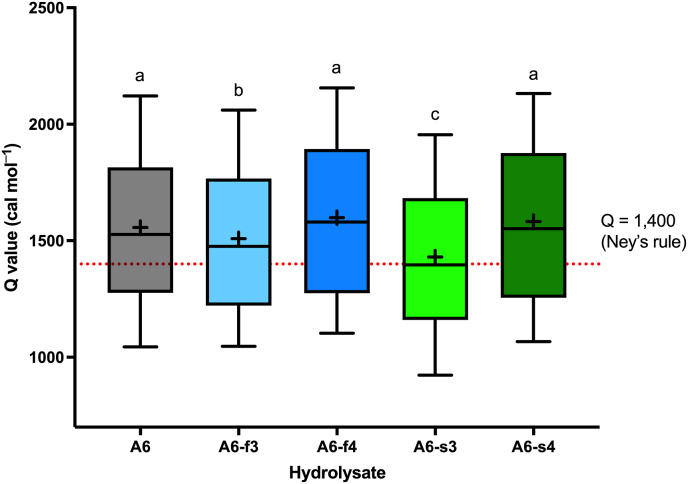
Q value distributions for five hydrolysates. Box: 25th–75th percentile; line: median; +: mean; whiskers: 10th–90th percentile. Dashed line: Q = 1400 cal mol^−1^ (Ney's threshold). Different letters indicate significant differences (Kruskal–Wallis with Dunn's test, Bonferroni, p < 0.05). Peptides identified by LC-MS/MS de novo sequencing; I/L assignments are interchangeable. These exploratory peptide-level comparisons do not represent treatment-level biological replication.

LC-MS/MS de novo sequencing identified 963–1226 distinct peptide sequences per hydrolysate (Table 3). A6-s3 exhibited the lowest mean Q value (1430 cal mol^−1^) and was the only sample approaching the 1400 cal mol^−1^ threshold (Fig. 4). At the level of the peptide sets detected in the present DDA runs, the Q-value distributions differed significantly (Kruskal–Wallis H = 107.43, p = 2.57 × 10^−22^); this exploratory result does not represent treatment-level biological replication. Dunn's post-hoc test assigned A6-s3 to a distinct group (c), significantly lower than A6, A6-f4, and A6-s4 (group a), and A6-f3 (group b) (p < 0.001 for all pairwise comparisons vs. A6-s3).

**Table 3:** Peptide characteristics of rice protein hydrolysates identified by LC-MS/MS de novo sequencing.

Sample	Total peptides	Mean Q (cal mol^−1^)	Median Q (cal mol^−1^)	Predicted bitter peptides (%)	Peptides below the predicted bitterness threshold (%)	Mean length (aa)
**A6**	1010	1557	1540	63.27	36.73	7.5
**A6-f3**	1226	1509	1460	58.97	41.03	7.5
**A6-f4**	1127	1599	1580	66.02	33.98	6.9
**A6-s3**	963	1430	1396	49.64	50.36	7.8
**A6-s4**	1150	1583	1560	64.35	35.65	6.8

Bitter peptides defined as Q value > 1400 cal mol^−1^ (Ney, 1971).

Unique peptides: proportion of peptides found exclusively in A6-s3 and not shared with any other sample.

Mean length: average peptide chain length in amino acid residues.

**Fig. 5:**
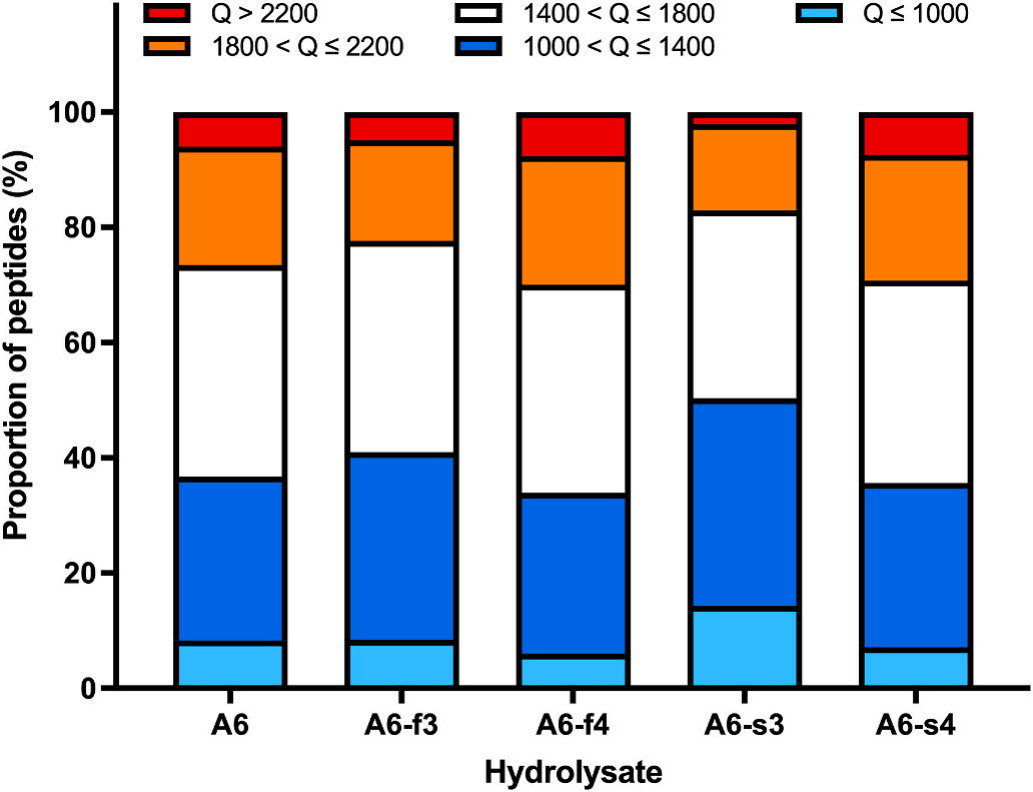
Proportion of peptides in five Q value categories. Predicted non-bitter: Q ≤ 1400 cal mol^−1^ (blue segments); predicted bitter: Q > 1400 cal mol^−1^ (white/orange/red segments). Classification based on Ney's rule and does not reflect sensory-confirmed bitterness of individual peptides.

Q value category analysis (Fig. 5) showed that A6-s3 had the highest proportion of peptides below the predicted bitterness threshold (Section 2.8; 50.4%) and the lowest proportion of highly hydrophobic peptides (Q > 2200 cal mol^−1^; 2.1%), compared with 36.7% and 6.0% for A6, respectively. A6-s3 also retained the numerically lowest abundance-weighted Q value (1461 cal mol^−1^; Table S1), although the value was close to that of A6-f3.

**Fig. 6:**
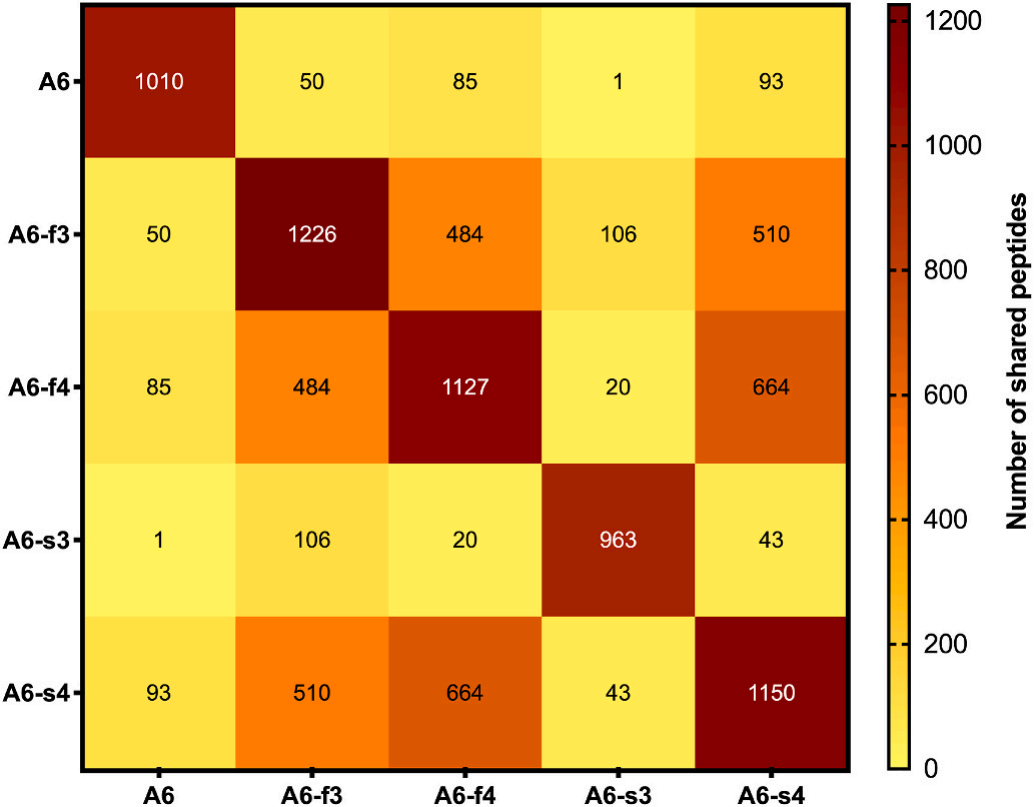
Peptide-overlap heatmap. Values indicate the number of shared peptides between each pair based on exact PEAKS-assigned sequence matches; diagonal values indicate the total number of detected peptides per sample. Because Q-TOF de novo sequencing cannot distinguish isoleucine from leucine, I/L assignments should be interpreted as interchangeable. Color intensity represents the number of shared peptides. A6 and A6-s3 shared one exact sequence (LPAF).

### 3.5 Peptide overlap, database search, and peptide-length analysis

The peptide-overlap heatmap (Fig. 6) showed that A6 and A6-s3 shared only one exact PEAKS-assigned sequence (LPAF) among >1900 combined detections. This low exact-sequence overlap indicates substantial differences in the peptides detected under the two conditions. However, exact matching does not capture nested parent–daughter relationships (e.g., ABCDEFG → BCDEFG is counted as different), and DDA acquisition is subject to stochastic precursor selection. The overlap result therefore cannot distinguish endopeptidase-mediated cleavage from progressive terminal processing. Database-assisted annotation provided an additional, but highly method-dependent, description of the detected peptide profiles (Dallas et al., 2015). In A6, 52.8% of detected peptides were assigned to the *Oryza sativa* proteome, with glutelins as the predominant sources (Table S6). A6-s3 showed a markedly lower database-assignment rate (0.2%) than the other hydrolysates (47.6–56.4%). This analytical observation accompanied substantial differences in the exact peptide sequences detected by DDA de novo sequencing, but it was not treated as independent evidence of peptide-profile remodeling or of a distinct cleavage mechanism. Proteolytic products necessarily remain contiguous subsequences of precursor proteins; the low assignment rate therefore does not imply non-native sequences. Potential contributors include non-specific cleavage, limited sequence uniqueness, I/L ambiguity, de novo sequencing uncertainty, unmodeled modifications, stochastic DDA precursor selection, and restricted coverage of the reviewed rice proteome.

Maintenance of pH 6.0 may have sustained Flavourzyme activity and contributed to the observed differences in the detectable peptide profile relative to free-fall treatment, in which pH gradually declined. Studies of plant and animal protein hydrolysates similarly show that bitterness depends on peptidase specificity, peptide-size distribution, and matrix composition rather than hydrolysis degree alone (Groβmann et al., 2021; Fan et al., 2019; Xia et al., 2022).

**Fig. 7:**
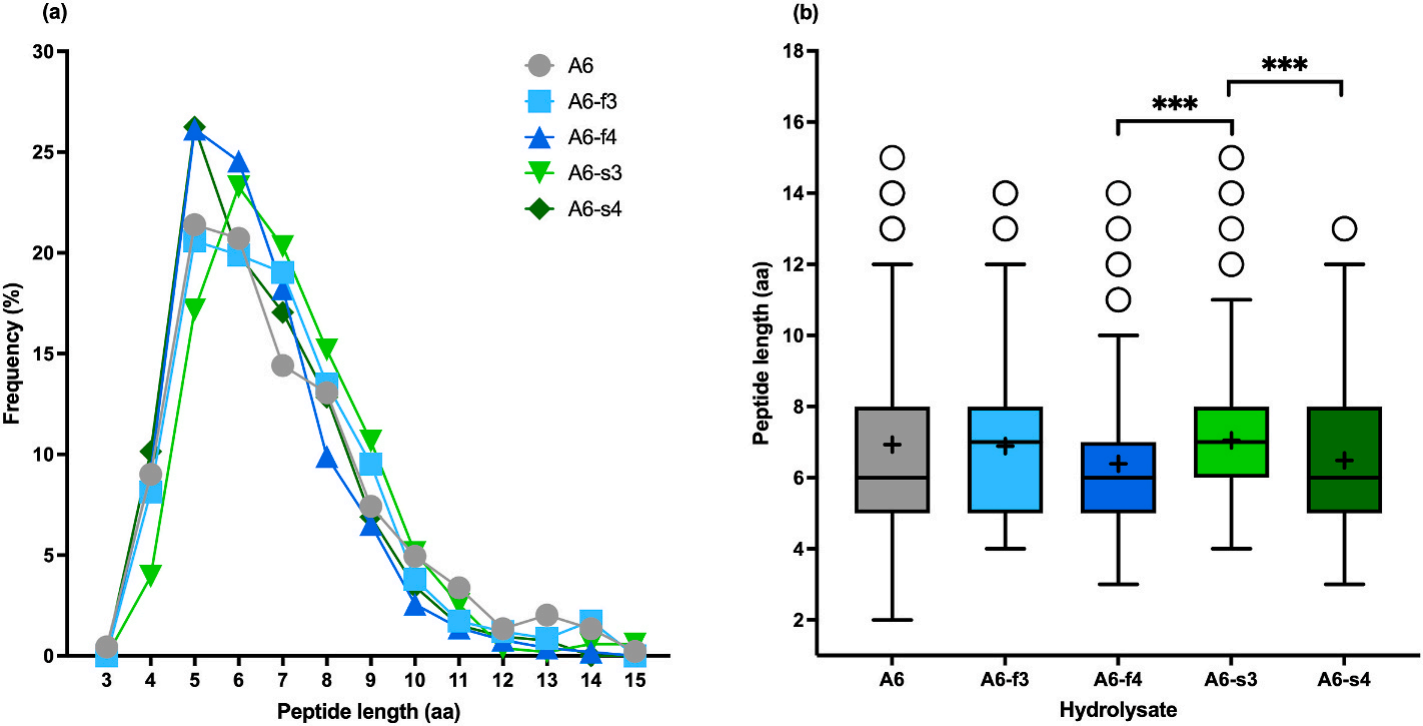
Peptide-length distributions of the five rice protein hydrolysates. (a) Frequency distribution of peptides by sequence length. (b) Box-and-whisker plots of peptide length. Boxes: interquartile range; horizontal lines: median; +: mean; whiskers: 1.5 × IQR; open circles: outliers. Sample sizes: A6, n = 1010; A6-f3, n = 1226; A6-f4, n = 1127; A6-s3, n = 963; A6-s4, n = 1150. Pairwise comparisons by Mann–Whitney *U* test with Bonferroni correction for 10 comparisons. Brackets indicate Bonferroni-adjusted pairwise comparisons involving A6-s3. ***p < 0.001. These exploratory peptide-level comparisons do not represent treatment-level biological replication.

Several additional observations support a more favorable bitterness-related profile for A6-s3. Peptides of 6–10 residues accounted for 74% of A6-s3 detections, whereas 2–5-residue peptides accounted for 21%, the lowest proportion among the five hydrolysates (Fig. 7). Mean peptide length was significantly higher than in A6-f4 and A6-s4 after Bonferroni correction, whereas differences from A6 and A6-f3 were not significant. These findings argue against accumulation of very short peptide tags as the sole explanation for the A6-s3 profile. Peptide length alone, however, neither determines sensory bitterness nor distinguishes internal from terminal cleavage and must be interpreted together with sequence hydrophobicity and terminal-residue characteristics. A6-s3 also showed the lowest C-terminal strongly hydrophobic residue frequency (62.3%; Table S3) and the highest N-terminal Shannon entropy (3.91 bits), consistent with greater diversity among the detected N termini.

Extending pH-stat hydrolysis from 3 to 4 h produced a higher mean Q value (1583 vs. 1430 cal mol^−1^) and a larger proportion of 2–5-residue peptides (36% vs. 21%). Previous studies have shown that extensive hydrolysis can increase bitterness by generating additional short hydrophobic peptides, although the relationship is enzyme- and matrix-dependent (FitzGerald and O'Cuinn, 2006; Fan et al., 2019; Xia et al., 2022). These results suggest that the favorable peptide-profile window may be relatively narrow under the tested conditions; nevertheless, A6-s3 and A6-s4 did not differ significantly in sensory bitterness after multiplicity correction.

The relative total amino acid profiles of the five hydrolysates were broadly comparable on descriptive inspection (Table S2), as expected from their common rice-protein starting material. Because the analysis measured amino acids after complete hydrolysis rather than free amino acids, it cannot determine whether Flavourzyme treatment changed the free-amino-acid pool and is presented only as descriptive compositional background. Table S10 lists representative high-abundance predicted bitter peptide sequences together with their Q values, sequence lengths, ALC scores, and semiquantitative LC-MS/MS peak areas.

### 3.6 Limitations

Although Ney's Q value provides a useful hydrophobicity-based index for comparing predicted bitterness potential across peptide populations, bitterness perception is not governed by hydrophobicity alone. Peptide length, terminal residue identity, charge distribution, sequence context, molecular-weight distribution, and matrix interactions may all modulate sensory bitterness (Fan et al., 2019; Xia et al., 2022; Maehashi and Huang, 2009; Ishibashi et al., 1988). Moreover, LC-MS/MS peak areas provide semi-quantitative abundance estimates that may be affected by ionization efficiency differences among peptides. The Q-value analysis was therefore interpreted as a comparative peptidomic indicator that complemented, rather than replaced, the sensory evaluation. The low exact-sequence overlap and near-zero database-assignment rate in A6-s3 indicate extensive differences in the detectable peptide profiles but cannot by themselves distinguish endopeptidase-mediated cleavage from progressive terminal trimming, owing to exact-match limitations (nested parent–daughter peptides are counted as “different”), I/L ambiguity, stochastic DDA precursor selection, and the absence of biological replicates for the LC-MS/MS analysis. The present population-level peptidomic data therefore do not permit quantitative separation of the relative contributions of Flavourzyme's endo- and exopeptidase activities. Additionally, the preliminary and formal sensory evaluations differed in panel size, methodology, and ultrafiltration status; consequently, the bitterness reversal cannot be attributed exclusively to ultrafiltration and is interpreted as a working hypothesis. Lyophilization and reconstitution may have caused selective loss of highly hydrophobic peptides through adsorption or aggregation. Although all samples underwent the same procedure, sample-dependent loss cannot be excluded because their peptide compositions differed, and absolute peptide recovery was not assessed. Ultrafiltration recovery rates were not determined; differential adsorption of hydrophobic peptides onto the membrane cannot be excluded and may have contributed to the observed differences among the ultrafiltered fractions.

### 3.7 Practical implications

From an application perspective, A6-s3 maintained high soluble nitrogen (Kjeldahl solubility 77.87%) and showed the most favorable overall combination of bitterness-related peptidomic indicators among the tested conditions. Because the sensory and peptidomic comparisons were conducted using the <2500 Da permeate, the practical performance of the complete, non-fractionated hydrolysate remains to be validated before industrial application. The pH-stat mode requires continuous NaOH titration, which adds process complexity and may increase sodium content; this should be considered during scale-up. DH-OPA (37.14%) and soluble solids (5.80 °Brix) may serve as candidate process-control indicators, but the proposed 3-h condition requires further validation. Several approaches would strengthen the findings: (1) electronic-tongue or taste-dilution assays could complement human sensory evaluation, as demonstrated in debittering studies of plant protein hydrolysates (Xia et al., 2022); (2) DIA-based peptidomics with biological replicates could improve quantitative coverage and reproducibility relative to the present single-run DDA design (Gotti et al., 2021); (3) activity-guided fractionation followed by sensory confirmation and MS identification would be required to establish the bitterness of individual sequences (Liu et al., 2014); and (4) parallel LC-MS/MS profiling of unfiltered hydrolysates and their ultrafiltration retentate and permeate fractions would clarify the basis of the apparent ranking difference between preliminary and formal sensory evaluations.

## 4 Conclusions

Under the conditions examined, 3 h of pH-stat Flavourzyme treatment produced an ultrafiltered fraction located at the least-bitter end of the sensory ranking and characterized by the lowest mean Q value (1430 cal mol^−1^), the lowest predicted bitter peptide proportion (49.6%), a reduced C-terminal strongly hydrophobic residue frequency (62.3% vs. 72.0% in A6), and the highest proportion of 6–10-residue peptides (74%). Its sensory bitterness did not differ significantly from A6-s4 after multiplicity correction. Low exact-sequence overlap with A6 and a markedly lower database-assignment rate indicate extensive differences in the detectable peptide profiles, but these observations are subject to the limitations of exact matching, I/L ambiguity, stochastic DDA sampling, and database coverage. Overall, the findings are compatible with established terminal processing accompanied by broader treatment-associated changes in the detectable peptide profile; they do not establish or quantify a distinct contribution from internal cleavage. DIA-based peptidomics with biological replicates, activity-guided validation of individual bitter peptides, and complementary sensory technologies are needed before translation to industrial debittering processes.

## CRediT authorship contribution statement

Pei-Yu Wu: Methodology, Validation, Writing – original draft. Yu-Shun Lin: Resources, Formal analysis, Data curation. Mei-Ling Li: Methodology, Data curation. Yun-Hsuan Chien: Investigation, Methodology, Data curation. Shang-Ming Huang: Supervision, Writing – review & editing. Kuo-Chiang Hsu: Conceptualization, Supervision, Writing – review & editing, Funding acquisition.

## Ethics declaration

Written informed consent to take part in the study and to publish the article has been obtained from all participants or their legal representatives. The privacy rights of participants have been observed.

This study was performed in compliance with relevant laws, regulatory frameworks and guidelines where the research took place. This study was conducted in accordance with Declaration of Helsinki. This study was approved by the Research Ethics Committee, China Medical University and Hospital. (Approval No. CMUH114-REC1-034).

## Consent for publication

Not applicable.

## Declaration of generative AI and AI-assisted technologies in the writing process

During preparation of this work, the authors used Claude (Anthropic) to assist with drafting analysis scripts, checking statistical workflows, generating preliminary figure code, and improving manuscript language. All analyses were executed, reviewed, and independently verified by the authors, who take full responsibility for the data, interpretation, and content of the publication.

## Funding

This work was supported by 10.13039/501100012544China Medical University, Taiwan (Grant No. CMU114-MF-38).

## Declaration of competing interests

The authors declare that they have no known competing financial interests or personal relationships that could have appeared to influence the work reported in this paper.

## Data Availability

The complete peptide-level dataset is provided as Dataset S1. Additional data supporting the findings of this study are available from the corresponding author upon reasonable request.
