# Supplementary material for: Peptidomic evidence for pH-stat flavourzyme-mediated debittering of Alcalase-derived rice protein hydrolysates
Source: Curr Res Food Sci. 2026 Aug 10;13:101529. doi: 10.1016/j.crfs.2026.101529 (PMC13487615; doi:10.1016/j.crfs.2026.101529)
Supplement: Supplementary file 1 [file mmc2.docx]

**Supplementary Material**

*Peptidomic evidence for pH-stat Flavourzyme-mediated debittering of Alcalase-derived rice protein hydrolysates*

**Figure S1.** Example ballot used for formal paired-comparison bitterness evaluation.

The ballot illustrates the response format used in the formal paired-comparison test. Actual sample identities were blinded using three-digit random codes, and the serving order was randomized and balanced across panelists.

**Sensory evaluation of bitterness difference between paired rice protein hydrolysates**

| **Panelist ID:** | ____________ | **Date:** | ____________ |
| --- | --- | --- | --- |
| **Session:** | □ 1 □ 2 | **Page:** | ____________ |

Instructions: For each pair, taste the two coded samples in the order shown. Hold each sample in the mouth for approximately 5 s, then expectorate. Rinse with distilled water and eat an unsalted cracker between pairs. Indicate which sample is more bitter and mark the degree of bitterness difference on the 0-7 scale.

| **Pair** | **First sample code** | **Second sample code** | **Which sample is more bitter?** | **Bitterness difference score** |
| --- | --- | --- | --- | --- |
| 1 | ___ | ___ | □ First sample □ Second sample □ No difference | 0 1 2 3 4 5 6 7 |
| 2 | ___ | ___ | □ First sample □ Second sample □ No difference | 0 1 2 3 4 5 6 7 |
| 3 | ___ | ___ | □ First sample □ Second sample □ No difference | 0 1 2 3 4 5 6 7 |
| 4 | ___ | ___ | □ First sample □ Second sample □ No difference | 0 1 2 3 4 5 6 7 |
| 5 | ___ | ___ | □ First sample □ Second sample □ No difference | 0 1 2 3 4 5 6 7 |

**Scale definition for the bitterness difference score.**

| **Score** | **Definition** |
| --- | --- |
| 0 | No difference |
| 1 | Very slight difference |
| 2 | Slight difference |
| 3 | Moderate difference |
| 4 | Moderately strong difference |
| 5 | Strong difference |
| 6 | Very strong difference |
| 7 | Extremely strong difference |

**Table S1.** Comparison of simple mean and abundance-weighted Q values of rice protein hydrolysates.

| **Sample** | **N** | **Simple mean Q**  **(cal mol^-1^)** | **Weighted Q (cal mol^-1^)** | **ΔQ**  **(cal mol^-1^)** | **Pred. bitter**  **% (count)** | **Pred. bitter**  **% (area)** | **ΔBitter**  **(%)** |
| --- | --- | --- | --- | --- | --- | --- | --- |
| **A6** | 1010 | 1556.8 | 1532.0 | −24.9 | 63.3 | 56.3 | −6.9 |
| **A6-f3** | 1226 | 1508.9 | 1469.8 | −39.1 | 59.0 | 60.0 | +1.0 |
| **A6-f4** | 1127 | 1599.2 | 1617.7 | +18.6 | 66.0 | 68.5 | +2.5 |
| **A6-s3** | 963 | 1430.2 | 1461.4 | +31.2 | 49.6 | 51.2 | +1.5 |
| **A6-s4** | 1150 | 1582.7 | 1527.3 | −55.5 | 64.3 | 62.0 | −2.4 |

Abundance-weighted Q value = Σ(Q_i_ × Area_i_) / Σ(Area_i_), where Q_i_ is the Q value and Areai is the LC-MS/MS peak area.

ΔQ = abundance-weighted Q − simple mean Q. ΔBitter = area-weighted bitter proportion − count-based bitter proportion.

Predicted bitter peptides were defined as Q > 1,400 cal mol-1 according to Ney’s criterion [15].

**Table S2.** Total amino acid composition of rice protein hydrolysates (g kg^-1^).

| **Amino acid** | **A6** | **A6-f3** | **A6-f4** | **A6-s3** | **A6-s4** |
| --- | --- | --- | --- | --- | --- |
| **Histidine (H)** | 20.43 | 18.93 | 17.32 | 17.58 | 18.42 |
| **Arginine (R)** | 72.39 | 63.85 | 56.94 | 63.25 | 66.28 |
| **Methionine (M)** | 20.07 | 18.40 | 17.09 | 17.63 | 18.58 |
| **Valine (V)** | 50.66 | 47.98 | 45.87 | 46.89 | 48.57 |
| **Leucine (L)** | 70.65 | 62.96 | 59.20 | 61.40 | 63.89 |
| **Phenylalanine (F)** | 42.42 | 39.33 | 36.83 | 38.01 | 39.79 |
| **Tyrosine (Y)** | 37.62 | 33.21 | 30.60 | 31.14 | 33.44 |
| **Isoleucine (I)** | 34.93 | 33.88 | 32.11 | 32.35 | 33.36 |
| **Tryptophan (W)** | 13.83 | 12.96 | 13.16 | 13.09 | 12.81 |
| **Glycine (G)** | 36.96 | 33.31 | 31.02 | 31.62 | 32.72 |
| **Serine (S)** | 40.40 | 38.87 | 33.00 | 35.89 | 36.54 |
| **Threonine (T)** | 29.57 | 27.98 | 25.27 | 26.09 | 27.68 |
| **Aspartic acid (D)** | 56.71 | 52.73 | 49.05 | 50.78 | 49.87 |
| **Glutamic acid (E)** | 115.37 | 106.75 | 99.56 | 106.49 | 108.35 |
| **Alanine (A)** | 53.54 | 47.74 | 44.25 | 46.08 | 48.57 |
| **Lysine (K)** | 32.50 | 27.94 | 26.73 | 27.99 | 29.05 |
| **Proline (P)** | 39.57 | 27.19 | 23.22 | 25.10 | 28.62 |
| **Cysteine (C)** | 5.30 | 5.64 | 5.39 | 5.19 | 4.13 |
| **Total** | 409.91 | 368.14 | 337.48 | 355.22 | 365.51 |

Values represent a single determination of total amino acid composition performed by a contracted laboratory. Amino acids other than tryptophan were determined using an in-house method based on AOAC 994.12 (Amino Acids in Feeds), whereas tryptophan was determined using an in-house method based on AOAC 988.15 (Tryptophan in Foods and Feed Ingredients). Because only one determination was available for each sample, the data are interpreted descriptively. Amino acids classified as bitter according to the selected Δf criterion (H, R, M, V, L, F, Y, I, and W; side-chain Δf > 500 cal mol^−1^) accounted for 46.96–47.83% of the total amino acid composition on a molar basis. This narrow range is consistent with the common rice-protein starting material and is presented as descriptive compositional information rather than evidence of free-amino-acid accumulation or sensory bitterness.

A6, Alcalase hydrolysate (6 h); f, free-fall pH; s, pH-stat; 3 and 4, Flavourzyme hydrolysis duration (h).

**Table S3.** Terminal amino acid characteristics and diversity of rice protein hydrolysates.

| **Parameter** | **A6** | **A6-f3** | **A6-f4** | **A6-s3** | **A6-s4** |
| --- | --- | --- | --- | --- | --- |
| **N-terminal hydrophobic (%)** | 52.7 | 57.0 | 58.9 | 54.2 | 57.7 |
| **N-terminal strong hydrophobic (%)** | 43.9 | 42.7 | 45.2 | 40.8 | 43.3 |
| **C-terminal hydrophobic (%)** | 83.5 | 78.8 | 79.9 | 79.2 | 79.7 |
| **C-terminal strong hydrophobic (%)** | 72.0 | 64.6 | 67.3 | 62.3 | 67.2 |
| **Both terminals hydrophobic (%)** | 43.5 | 44.9 | 47.9 | 42.9 | 47.3 |
| **Neither terminal hydrophobic (%)** | 7.3 | 9.1 | 9.1 | 9.4 | 9.8 |
| **N-terminal Shannon entropy (bits)** | 2.844 | 3.671 | 3.651 | 3.913 | 3.686 |
| **C-terminal Shannon entropy (bits)** | 2.452 | 2.556 | 2.520 | 2.591 | 2.501 |

Higher-Δf residues (Ney/Tanford scale): V, I, K, L, M, F, Y, W, P (side-chain Δf ≥ 1,300 cal mol^-1^). Strongly hydrophobic residues: I, L, F, W, Y, P (side-chain Δf ≥ 2,400 cal mol^-1^).

Shannon entropy (H) = −Σ(p_i_ × log2 p_i_), where p_i_ is the frequency of amino acid i at the terminal position. Higher entropy indicates greater diversity.

A6-s3 exhibited the lowest C-terminal strong hydrophobic proportion (62.3%) and the highest N-terminal Shannon entropy (3.913 bits).

**Table S4.** Degree of hydrolysis (DH), protein solubility, and soluble solids of rice protein hydrolysates during first-stage hydrolysis with three proteases.

| **Enzyme** | **Time (h)** | **DH-OPA (%)** | **DH-pH-stat (%)** | **DH-TNBS (%)** | **Solubility-Lowry (%)** | **Solubility-Kjeldahl (%)** | **Soluble solids (°Brix)** |
| --- | --- | --- | --- | --- | --- | --- | --- |
| **Alcalase** | 0 | 0 | 0 | 0 | 2.38 ± 0.61 | 3.03 ± 2.36 | 0.20 ± 0.00 |
|  | 1 | 17.84 ± 1.82 | 8.18 ± 0.61 | 14.14 ± 1.00 | 62.35 ± 1.42 | 65.86 ± 2.93 | 3.90 ± 0.14 |
|  | 2 | 18.92 ± 0.93 | 10.27 ± 0.31 | 18.34 ± 0.63 | 61.65 ± 2.07 | 69.20 ± 2.78 | 4.20 ± 0.20 |
|  | 3 | 21.13 ± 0.35 | 12.40 ± 0.30 | 22.53 ± 0.42 | 62.59 ± 0.94 | 71.81 ± 2.18 | 4.60 ± 0.00 |
|  | 4 | 22.50 ± 0.57 | 14.47 ± 0.48 | 25.28 ± 0.60 | 63.45 ± 1.38 | 74.52 ± 3.19 | 4.80 ± 0.00 |
|  | 5 | 23.83 ± 0.45 | 17.01 ± 0.42 | 27.53 ± 0.71 | 66.11 ± 2.29 | 75.89 ± 5.25 | 5.00 ± 0.00 |
|  | 6 | 24.69 ± 0.77 | 19.39 ± 0.72 | 30.65 ± 0.53 | 69.10 ± 1.37 | 78.12 ± 3.02 | 5.13 ± 0.12 |
| **Papain** | 0 | 0 | 0 | 0 | 1.38 ± 0.58 | 2.58 ± 2.75 | 0.00 ± 0.00 |
|  | 1 | 3.24 ± 0.16 | 1.83 ± 0.09 | 4.06 ± 0.56 | 25.03 ± 0.06 | 22.63 ± 1.73 | 1.60 ± 0.20 |
|  | 2 | 4.05 ± 0.29 | 2.19 ± 0.10 | 4.76 ± 0.35 | 22.83 ± 0.84 | 24.06 ± 2.57 | 1.60 ± 0.00 |
|  | 3 | 3.91 ± 0.16 | 2.37 ± 0.24 | 5.11 ± 0.28 | 26.86 ± 0.58 | 29.12 ± 2.47 | 1.73 ± 0.12 |
|  | 4 | 3.89 ± 0.32 | 2.74 ± 0.24 | 5.54 ± 0.43 | 25.99 ± 1.81 | 29.89 ± 3.17 | 1.80 ± 0.00 |
|  | 5 | 4.10 ± 0.18 | 3.14 ± 0.11 | 6.44 ± 0.19 | 29.02 ± 1.43 | 30.66 ± 3.15 | 1.87 ± 0.12 |
|  | 6 | 4.31 ± 0.07 | 3.51 ± 0.18 | 7.00 ± 0.21 | 29.11 ± 1.43 | 32.39 ± 4.09 | 1.93 ± 0.12 |
| **Flavourzyme** | 0 | 0 | 0 | 0 | 0.88 ± 0.00 | 0.89 ± 1.21 | 0.00 ± 0.00 |
|  | 1 | 7.51 ± 1.73 | 0.76 ± 0.16 | 3.26 ± 0.69 | 5.94 ± 1.07 | 13.53 ± 3.20 | 0.93 ± 0.12 |
|  | 2 | 9.07 ± 0.74 | 1.48 ± 0.11 | 5.79 ± 0.47 | 13.07 ± 0.91 | 16.11 ± 2.38 | 1.30 ± 0.14 |
|  | 3 | 10.87 ± 1.37 | 2.06 ± 0.07 | 7.52 ± 0.46 | 14.67 ± 1.56 | 18.84 ± 2.99 | 1.40 ± 0.20 |
|  | 4 | 13.00 ± 2.21 | 2.95 ± 0.16 | 10.51 ± 0.80 | 16.32 ± 0.32 | 22.51 ± 5.59 | 1.70 ± 0.14 |
|  | 5 | 13.88 ± 1.34 | 3.87 ± 0.04 | 12.61 ± 0.59 | 18.51 ± 0.50 | 22.91 ± 3.43 | 1.80 ± 0.20 |
|  | 6 | 13.95 ± 2.14 | 4.50 ± 0.43 | 14.40 ± 0.70 | 20.72 ± 1.10 | 25.83 ± 2.77 | 2.00 ± 0.00 |

Values are mean ± SD (n = 3). Hydrolysis conditions: Alcalase (pH 9, 55 °C), Papain (pH 7, 50 °C), Flavourzyme (pH 6, 60 °C). E/S = 5% (w/w). pH-stat method.

**Table S5.** Sensitivity analysis: effect of ALC score cutoff on Q value statistics and peptide overlap.

| **ALC cutoff** | **N (A6)** | **N**  **(A6-s3)** | **Mean Q A6-s3** | **Pred. bitter % A6-s3** | **A6-s3**  **rank** | **Shared (A6/A6-s3)** | **KW**  **p-value** | **Dunn's**  **A6-s3 vs all** |
| --- | --- | --- | --- | --- | --- | --- | --- | --- |
| ≥75% (all) | 1010 | 963 | 1430 | 49.6 | 1st (lowest) | 1 | 2.57 × 10^–22^ | *p* < 0.001 |
| ≥80% | 1010 | 639 | 1437 | 49.8 | 1st (lowest) | 1 | 5.04 × 10^–16^ | *p* < 0.01 |
| ≥90% | 441 | 166 | 1457 | 53.0 | 1st (lowest) | 0 | — | — |

N (A6) and N (A6-s3): number of peptides retained at each ALC cutoff. Mean Q and predicted bitter peptide proportion: values for A6-s3. Shared: number of peptides in common between A6 and A6-s3.

A6-s3 retained the lowest mean Q value and the lowest predicted bitter peptide proportion at all ALC cutoffs. The peptide overlap between A6 and A6-s3 remained ≤1 at all cutoffs.

KW: Kruskal–Wallis H test comparing Q value distributions among all five hydrolysates. Dunn’s: Bonferroni-corrected post-hoc comparison of A6-s3 vs. each other group.

**Table S6.** Database search validation: proportion of de novo-identified peptides matched to the *Oryza sativa* proteome (UniProt/SwissProt).

| **Sample** | **Total peptides** | **Matched (%)** | **Unmatched (%)** | **Glutelin peptides** | **Top matched proteins** |
| --- | --- | --- | --- | --- | --- |
| A6 | 1010 | 533 (52.8) | 477 (47.2) | 159 | GLUB4, GLUB5, GLUB1, GLUA2, GLUB2, GLUD1, GLUA1 |
| A6-f3 | 1226 | 583 (47.6) | 643 (52.4) | 0 | MSL1, RA05, DCL1, BIG, XIAO |
| A6-f4 | 1127 | 636 (56.4) | 491 (43.6) | 89 | GLUA2, GLUA1, SSG1, ILL1, GLUB1 |
| A6-s3 | 963 | 2 (0.2) | 961 (99.8) | 0 | — |
| A6-s4 | 1150 | 638 (55.5) | 512 (44.5) | 78 | GLUA2, GLUA1, PROA, TOR, ILL1 |

Matched: peptides with at least one protein accession identified by PEAKS database search against the Oryza sativa proteome (UniProt/SwissProt).

Glutelin peptides: number of matched peptides mapping to glutelin proteins (GLUA1, GLUA2, GLUA3, GLUB1, GLUB2, GLUB4, GLUB5, GLUD1).

A6-s3 showed a markedly lower database-assignment rate (0.2%) than the other hydrolysates. This result should be interpreted cautiously because database-assignment rates may be influenced by peptide length, sequence uniqueness, I/L ambiguity, de novo sequencing uncertainty, non-specific cleavage, possible modifications, stochastic DDA precursor selection, search parameters, and reference-database coverage. Accordingly, the database-assignment rate is treated as a descriptive analytical observation rather than direct mechanistic evidence.

In A6, many database-assigned peptides mapped to expected rice protein sources, particularly glutelins.

**Table S7.** Design of sensory evaluation and panel training.

| **Item** | **Description** |
| --- | --- |
| **Panelists** | Fifteen trained panelists participated in the formal sensory evaluation. |
| **Preliminary screening** | A preliminary bitterness screening was conducted to select hydrolysis time points for formal evaluation. A6 was used as the most bitter reference and distilled water as the non-bitter reference. The preliminary screening was used only for time-point selection and not for the final bitterness ranking. |
| **Panel training** | Panelists were trained before the formal test to recognize bitterness intensity, use the paired-comparison ballot, and apply the 0–7 bitterness difference scale. |
| **Formal samples** | A6, A6-f3, A6-f4, A6-s3, and A6-s4. |
| **Sample preparation** | Hydrolysates were ultrafiltered through a 2,500 Da MWCO membrane, freeze-dried, and reconstituted to equivalent nitrogen concentration before sensory evaluation. |
| **Evaluation design** | Paired-comparison design using all 10 pairwise combinations among the five hydrolysates. |
| **Sessions** | The formal evaluation was conducted in two sessions. |
| **Comparisons per panelist** | Each panelist evaluated five pairs per session, giving 10 pairwise comparisons in total. |
| **Serving order** | Pair order was randomized, and left/right serving positions were balanced. Samples were labeled with three-digit random codes. |
| **Evaluation procedure** | For each pair, panelists tasted the two coded samples in the order shown on the ballot. Each sample was held in the mouth for approximately 5 s before expectoration. |
| **Palate cleansing** | Panelists rinsed their mouth with distilled water and consumed an unsalted cracker between samples or pairs. A rest interval of at least 30 s was used between pairs. |
| **Response format** | Panelists indicated which sample in each pair was more bitter and rated the degree of bitterness difference on a 0–7 scale. |
| **Scale definition** | 0 = no difference; 1 = very slight difference; 2 = slight difference; 3 = moderate difference; 4 = moderately strong difference; 5 = strong difference; 6 = very strong difference; 7 = extremely strong difference. |
| **Statistical analysis** | Pairwise comparisons were analyzed using the Wilcoxon signed-rank test for difference-score data or the sign test for direction-only data, with Bonferroni correction for 10 comparisons. |

**Table S8.** Detailed statistical results of the formal paired-comparison sensory evaluation.

| **Comparison** | **More bitter sample** | **Mean difference score** | **Test used** | **Raw *p* value** | **Bonferroni-adjusted *p* value** | **Interpretation** |
| --- | --- | --- | --- | --- | --- | --- |
| A6 vs A6-f3 | A6 | 3.75 | Wilcoxon signed-rank test | < 0.001 | < 0.01 | A6 significantly more bitter |
| A6 vs A6-f4 | A6 | 2.78 | Wilcoxon signed-rank test | < 0.001 | < 0.01 | A6 significantly more bitter |
| A6 vs A6-s3 | A6 | 3.64 | Wilcoxon signed-rank test | < 0.001 | < 0.01 | A6 significantly more bitter |
| A6 vs A6-s4 | A6 | 4.03 | Wilcoxon signed-rank test | < 0.001 | < 0.01 | A6 significantly more bitter |
| A6-f3 vs A6-f4 | A6-f4 | 3.33 | Wilcoxon signed-rank test | < 0.001 | < 0.01 | A6-f4 significantly more bitter |
| A6-f3 vs A6-s3 | A6-f3 | 1.68 | Wilcoxon signed-rank test | 0.003 | < 0.05 | A6-f3 significantly more bitter |
| A6-f3 vs A6-s4 | — | — | Sign test | > 0.05 | > 0.05 | Not significant |
| A6-f4 vs A6-s3 | A6-f4 | 2.75 | Wilcoxon signed-rank test | < 0.001 | < 0.01 | A6-f4 significantly more bitter |
| A6-f4 vs A6-s4 | A6-f4 | 2.13 | Wilcoxon signed-rank test | 0.002 | < 0.05 | A6-f4 significantly more bitter |
| A6-s3 vs A6-s4 | — | — | Sign test | > 0.05 | > 0.05 | Not significant |

**Table S9.** LC-MS/MS acquisition and PEAKS data analysis parameters.

| **Parameter** | **Setting** |
| --- | --- |
| **Liquid chromatography** |  |
| System | UltiMate 3000 RSLCnano (Dionex) |
| Column | Acclaim PepMap C18 (75 μm × 250 mm, 2 μm, 100 Å; Thermo Scientific) |
| Mobile phase A | 0.1% formic acid in water |
| Mobile phase B | 0.1% formic acid in acetonitrile |
| Gradient | 8–40% B over 90 min (0.35%/min) |
| Flow rate | 300 nL min⁻¹ |
| **Mass spectrometry** |  |
| System | maXis Impact Q-TOF (Bruker Daltonics) |
| Ionization | ESI, positive ion mode |
| Scan range | m/z 150–1500 |
| Scan rate | 1 Hz (MS1), 10 Hz (MS2) |
| DDA | Top 8 precursor ions per cycle |
| Charge states | +2, +3, +4 |
| **Data analysis** |  |
| Software | PEAKS Studio 11.0 (Bioinformatics Solutions Inc.) |
| Parent mass tolerance | 50 ppm |
| Fragment mass tolerance | 0.05 Da |
| ALC score cutoff | ≥75% |
| Fixed modification | Carbamidomethylation (C) |
| Variable modifications | Oxidation (M); Deamidation (NQ) |
| Database | UniProt/SwissProt Oryza sativa (accessed June 2024) |
| Enzyme specificity | Unspecific cleavage |
| Match criterion | At least one Oryza sativa protein accession assigned by PEAKS |
| I/L ambiguity | Not distinguished; all assignments interchangeable |

Analysis was performed at the Proteomics Core Laboratory, China Medical University.

**Table S10.** Top 10 most abundant predicted bitter peptides (Q > 1,400 cal mol^−1^) in each hydrolysate, ranked by LC-MS/MS peak area.

**A6** (1010 peptides; 639 predicted bitter)

| **Rank** | **Sequence** | **Length (aa)** | **Q (cal mol^-1^)** | **ALC (%)** | **Area (×10⁶) [Rel. %]** | **C-term** |
| --- | --- | --- | --- | --- | --- | --- |
| 1 | AAPEFL | 6 | 1617 | 99.2 | 65.7 [2.5%] | L ● |
| 2 | AAVPEKLQLLP | 11 | 1609 | 98.8 | 65.7 [2.5%] | P ● |
| 3 | FLLPGG | 6 | 1685 | 88.5 | 29.4 [1.1%] | G |
| 4 | FLLL | 4 | 2478 | 88.5 | 29.4 [1.1%] | L ● |
| 5 | EAVALFFL | 8 | 1730 | 93.4 | 28.6 [1.1%] | L ● |
| 6 | DLVAL | 5 | 1560 | 94.0 | 28.6 [1.1%] | L ● |
| 7 | FLVP | 4 | 2345 | 88.4 | 28.6 [1.1%] | P ● |
| 8 | AWPF | 4 | 2250 | 96.3 | 24.0 [0.9%] | F ● |
| 9 | CLLGPLL | 7 | 1757 | 96.1 | 24.0 [0.9%] | L ● |
| 10 | DHVLPYL | 7 | 1866 | 94.6 | 24.0 [0.9%] | L ● |

**A6-f3** (1226 peptides; 723 predicted bitter)

| **Rank** | **Sequence** | **Length (aa)** | **Q (cal mol^-1^)** | **ALC (%)** | **Area (×10⁶) [Rel. %]** | **C-term** |
| --- | --- | --- | --- | --- | --- | --- |
| 1 | YDNEWYG | 7 | 1404 | 82.4 | 62.1 [3.9%] | G |
| 2 | VPYNEP | 6 | 1725 | 82.9 | 62.1 [3.9%] | P ● |
| 3 | VDLPLGLRG | 9 | 1427 | 84.1 | 39.4 [2.5%] | G |
| 4 | PPPAPL | 6 | 2272 | 86.8 | 19.3 [1.2%] | L ● |
| 5 | LPDLDGKL | 8 | 1558 | 89.8 | 19.3 [1.2%] | L ● |
| 6 | SPLLWG | 6 | 1750 | 85.6 | 11.4 [0.7%] | G |
| 7 | LPLGGL | 6 | 1647 | 89.3 | 10.5 [0.7%] | L ● |
| 8 | VPGFF | 5 | 1922 | 83.3 | 10.5 [0.7%] | F ● |
| 9 | PSAPPKGF | 8 | 1598 | 86.8 | 8.0 [0.5%] | F ● |
| 10 | VPAMF | 5 | 1798 | 83.4 | 8.0 [0.5%] | F ● |

**A6-f4** (1127 peptides; 744 predicted bitter)

| **Rank** | **Sequence** | **Length (aa)** | **Q (cal mol^-1^)** | **ALC (%)** | **Area (×10⁶) [Rel. %]** | **C-term** |
| --- | --- | --- | --- | --- | --- | --- |
| 1 | LPSGF | 5 | 1546 | 94.5 | 32.5 [1.6%] | F ● |
| 2 | VDPVF | 5 | 1838 | 97.4 | 22.5 [1.1%] | F ● |
| 3 | FDRF | 4 | 1642 | 98.0 | 22.5 [1.1%] | F ● |
| 4 | LPLTA | 5 | 1726 | 94.4 | 22.2 [1.1%] | A |
| 5 | EESMPM(+15.99)LP | 8 | 1425 | 95.0 | 22.2 [1.1%] | P ● |
| 6 | DLFNHP | 6 | 1455 | 96.8 | 22.2 [1.1%] | P ● |
| 7 | APLYG | 5 | 1728 | 95.4 | 22.2 [1.1%] | G |
| 8 | SPTHLPRL | 8 | 1474 | 94.8 | 22.2 [1.1%] | L ● |
| 9 | FPLLNL | 6 | 2088 | 91.9 | 18.5 [0.9%] | L ● |
| 10 | FPLLM(+15.99) | 5 | 2282 | 93.9 | 18.5 [0.9%] | M |

**A6-s3** (963 peptides; 478 predicted bitter)

| **Rank** | **Sequence** | **Length (aa)** | **Q (cal mol^-1^)** | **ALC (%)** | **Area (×10⁶) [Rel. %]** | **C-term** |
| --- | --- | --- | --- | --- | --- | --- |
| 1 | DFFGPF | 6 | 1852 | 93.1 | 37.6 [2.2%] | F ● |
| 2 | LPKEM | 5 | 1678 | 83.4 | 34.8 [2.0%] | M |
| 3 | LN(+0.98)HPHPL | 7 | 1583 | 84.3 | 34.8 [2.0%] | L ● |
| 4 | DWFGAF | 6 | 1595 | 91.3 | 26.2 [1.5%] | F ● |
| 5 | LVM(+15.99)TM(+15.99) | 5 | 1430 | 82.1 | 26.2 [1.5%] | M |
| 6 | LM(+15.99)LL | 4 | 2140 | 84.8 | 26.2 [1.5%] | L ● |
| 7 | FLDQPM(+15.99) | 6 | 1588 | 88.7 | 26.2 [1.5%] | M |
| 8 | FPPTL | 5 | 2150 | 88.2 | 26.2 [1.5%] | L ● |
| 9 | PM(+15.99)YPEL | 6 | 2063 | 80.2 | 24.8 [1.4%] | L ● |
| 10 | VPYRRP | 6 | 1877 | 77.0 | 23.5 [1.3%] | P ● |

**A6-s4** (1150 peptides; 740 predicted bitter)

| **Rank** | **Sequence** | **Length (aa)** | **Q (cal mol^-1^)** | **ALC (%)** | **Area (×10⁶) [Rel. %]** | **C-term** |
| --- | --- | --- | --- | --- | --- | --- |
| 1 | LPHDPRL | 7 | 1693 | 92.6 | 27.0 [1.4%] | L ● |
| 2 | FEPLRGL | 7 | 1627 | 95.6 | 27.0 [1.4%] | L ● |
| 3 | KENVLPGKL | 9 | 1411 | 94.1 | 27.0 [1.4%] | L ● |
| 4 | FPLLM(+15.99) | 5 | 2282 | 95.1 | 27.0 [1.4%] | M |
| 5 | VDLPLGLG | 8 | 1514 | 88.5 | 21.2 [1.1%] | G |
| 6 | YTLM(+15.99) | 4 | 1758 | 86.0 | 21.2 [1.1%] | M |
| 7 | FFDSV | 5 | 1514 | 95.6 | 18.7 [1.0%] | V |
| 8 | DGQKFPLL | 8 | 1519 | 97.0 | 18.7 [1.0%] | L ● |
| 9 | MPVDVL | 6 | 1710 | 90.9 | 16.2 [0.8%] | L ● |
| 10 | ELLRP | 5 | 1748 | 96.2 | 16.2 [0.8%] | P ● |

Predicted bitter peptides: Q > 1,400 cal mol**^-1^** (Ney, 1971). Q = Σ(Δf)/n, where Δf is the side-chain free energy of transfer (Tanford, 1962).

Area: LC-MS/MS peak area from PEAKS de novo sequencing. Rel. %: peptide peak area expressed as a percentage of the summed peak area of all detected peptides in the corresponding sample. ALC: average local confidence score. C-term ●: strongly hydrophobic C-terminal residue (Δf ≥ 2,400 cal mol−1; I, L, F, W, Y, P). I/L ambiguity: Q-TOF de novo sequencing cannot distinguish isoleucine from leucine. Multiple de novo peptide assignments may originate from the same LC-MS feature and may therefore share identical peak-area values.

C-term ●: strongly hydrophobic C-terminal residue (Δf ≥ 2,400 cal mol**^-1^**; I, L, F, W, Y, P).

I/L ambiguity: Q-TOF de novo sequencing cannot distinguish isoleucine from leucine. Multiple de novo peptide assignments may originate from the same LC-MS feature and may therefore share identical peak-area values.

**Dataset S1. Complete peptide lists of five rice protein hydrolysates identified by LC-MS/MS de novo sequencing (separate Excel file).**

The dataset contains five worksheets (A6, A6-f3, A6-f4, A6-s3, A6-s4), each listing all peptides identified by de novo sequencing with the following columns: Peptide (amino acid sequence), Tag length (number of residues), Q Value (Ney’s bitterness prediction in cal mol^-1^), Bitter classification (Q > 1,400 cal mol^-1^), and Area (LC-MS/MS peak area for abundance weighting).

**Summary:**

A6: 1,010 peptides; A6-f3: 1,226 peptides; A6-f4: 1,127 peptides; A6-s3: 963 peptides; A6-s4: 1,150 peptides.
